# Mechanistic Pathways of Gestational Obesity: Implications for Maternal and Offspring Health: A Narrative Review

**DOI:** 10.3390/nu17233731

**Published:** 2025-11-28

**Authors:** Alireza Jahan-Mihan, Jamisha Leftwich, Corinne Labyak, Jill Snyder, Kristin Berg, Reniel R. Nodarse

**Affiliations:** Department of Nutrition and Dietetics, University of North Florida, 1 UNF Dr., Jacksonville, FL 32224, USA; j.leftwich@unf.edu (J.L.); c.labyak@unf.edu (C.L.); jill.snyder@unf.edu (J.S.); k.berg@unf.edu (K.B.); n01569362@unf.edu (R.R.N.)

**Keywords:** maternal obesity, fetal programming, offspring’s health, diet, mothers’ health

## Abstract

Gestational obesity, defined as obesity during pregnancy or a pre-pregnancy BMI ≥30, is a growing global health challenge with profound implications for both maternal and offspring health. This narrative review synthesizes current evidence on the mechanistic pathways by which maternal obesity affects pregnancy outcomes and intergenerational health trajectories. For mothers, gestational obesity increases the risk of gestational diabetes, hypertensive disorders, cesarean delivery, and postpartum weight retention. Offspring exposed to maternal obesity face higher risks of obesity, metabolic syndrome, cardiovascular disease, and neurodevelopmental disorders, many of which persist across the lifespan. The underlying mechanisms include metabolic dysregulation, insulin resistance, chronic inflammation, oxidative stress, and alterations in placental function. Epigenetic modifications, such as DNA methylation, histone changes, and non-coding RNA expression, play central roles in fetal programming, while maternal gut dysbiosis and alterations in breast milk microbiota further shape infant health outcomes. Importantly, maternal obesity not only influences pregnancy and early life but also perpetuates an intergenerational cycle of obesity and related comorbidities. Preventive strategies targeting preconception and prenatal health, combined with interventions to optimize lactation and maternal diet, may mitigate long-term risks. Future research should prioritize longitudinal and mechanistic studies to refine interventions aimed at disrupting the transmission of obesity-related disease across generations.

## 1. Introduction

The rising prevalence of obesity in women of reproductive age has elevated concern about the implications of gestational obesity, typically defined as obesity before conception or during pregnancy (e.g., BMI ≥ 30 kg/m^2^), on both maternal and offspring health. Recent data suggest that maternal obesity is not just a comorbid risk but a driver of long-term disease through developmental programming. For example, a recent systematic review found that maternal obesity is strongly associated with adverse maternal outcomes including gestational hypertension, preeclampsia, gestational diabetes mellitus (GDM), cesarean delivery, and postpartum weight retention [1,2,3].

From the offspring perspective, in utero exposure to maternal obesity has been linked to elevated risk of obesity, insulin resistance, metabolic syndrome, non-alcoholic fatty liver disease, and cardiovascular dysfunction later in life [3,4,5]. Emerging evidence also implicates cognitive and neurodevelopmental changes, as well as immune and inflammatory dysregulation, in offspring of obese pregnancies [5,6,7].

While epidemiological associations are well documented, understanding the mechanistic pathways is crucial to identifying intervention strategies. Several mechanistic themes have recently gained attention:

Maternal obesity defined as pre-pregnancy obesity promotes a range of biological disturbances that impact both the mother and offspring. Metabolic dysregulation, lipotoxicity, and oxidative stress are among the primary mechanisms, as elevated circulating lipids, glucose, and free fatty acids overwhelm fetal metabolic capacity. This excess burden leads to mitochondrial dysfunction, increased generation of reactive oxygen species, and subsequent cellular stress [4,8]. In addition, impaired mitochondrial quality control processes, such as autophagy and mitophagy, have been implicated in furthering metabolic and cellular dysfunction in the fetus and offspring of obese mothers [9].

Placental maladaptation and inflammation also play a critical role, with changes in placental vascular structure, heightened inflammation, and impaired nutrient transport linking maternal metabolic disturbances to fetal programming [3,4,7]. Alongside these changes, maternal obesity has been shown to induce epigenetic reprogramming through DNA methylation, histone modifications, and non-coding RNA expression. For example, hundreds of differentially methylated CpG sites have been identified in cord blood of offspring exposed to maternal obesity [4,9,10].

Another significant factor involves maternal–fetal microbiome interactions. The maternal gut and vaginal microbiome shape fetal and neonatal microbiota colonization, and obesity is frequently associated with dysbiosis, including alterations in the Firmicutes/Bacteroidetes ratio. These microbial shifts can modulate metabolic and immune programming through metabolites, inflammatory responses, and epigenetic pathways [4,5,11,12]. Finally, postnatal amplification and secondary challenges may further exacerbate risks, as offspring born to obese mothers often carry latent vulnerabilities that become unmasked when exposed to additional stressors such as high-fat diets, aging, or metabolic challenges later in life [4,9].

Given the complexity and interdependence of these pathways, a narrative integration of clinical outcomes and mechanistic evidence is needed. In this review, we synthesize recent findings on gestational obesity’s impact, focusing particularly on mechanistic underpinnings connecting maternal obesity with health trajectories in both mothers and offspring. We also highlight gaps and propose directions for future research that could inform preventive or therapeutic interventions to break the intergenerational cycle of obesity.

## 2. Methodology

This narrative review aimed to synthesize and critically evaluate current evidence on mechanistic pathways linking gestational obesity with maternal and offspring health outcomes. To ensure methodological transparency and reproducibility, a structured search strategy and a PRISMA-guided selection process were implemented. A comprehensive literature search was conducted across three major databases—PubMed, UNF OneSearch, and Google Scholar—covering the period from 1 January 2010 to 31 October 2024. The search strategy included combinations of keywords and Medical Subject Headings (MeSH), such as “pregnancy,” “maternal obesity,” “gestational obesity,” “fetal programming,” “developmental programming,” “maternal nutrition,” “epigenetics,” “placental function,” “oxidative stress,” “maternal microbiome,” and “gestational diabetes.” Boolean operators (AND, OR) were applied to refine results, and the reference lists of eligible studies and relevant reviews were hand-searched to identify additional sources.

Studies were included if they met predefined criteria regarding study design, population, and scientific focus. Eligible study designs included randomized controlled trials, cohort studies, case–control studies, experimental animal studies, and systematic reviews. Populations of interest included pregnant women with obesity (defined as pre-pregnancy or gestational BMI ≥ 30 kg/m^2^) and their offspring, as well as validated animal models of maternal obesity. Studies were required to address mechanistic pathways—such as inflammation, oxidative stress, metabolic dysregulation, placental alterations, epigenetic modifications, microbiome changes, or breastfeeding-related mechanisms—or long-term maternal or offspring outcomes tied to such pathways. Exclusion criteria included non-English publications, non-peer-reviewed or gray literature (e.g., conference abstracts, dissertations, commentaries), and studies not directly related to maternal obesity or mechanistic pathways.

**Figure 1:**
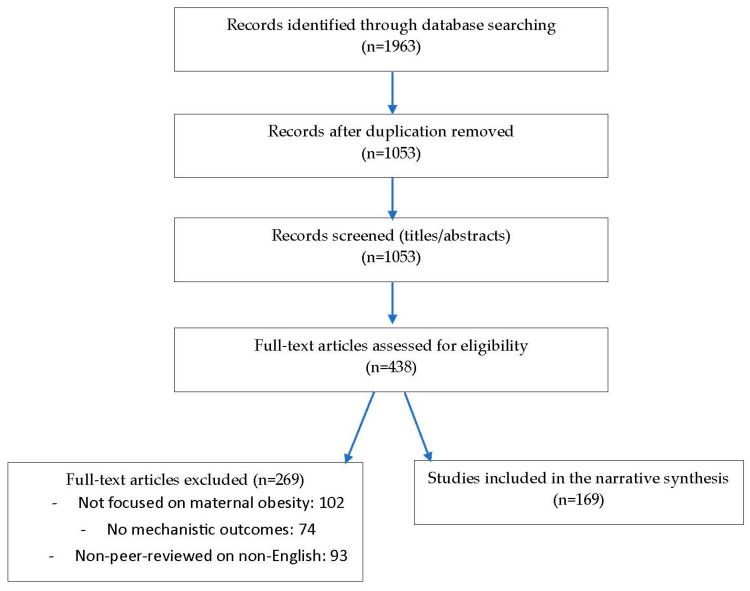
PRISMA Flow Diagram for paper selection process.

All search results were imported into a citation manager and underwent de-duplication. Two reviewers independently screened titles and abstracts, followed by full-text assessment of potentially relevant articles, with disagreements resolved in consultation with a third reviewer. A PRISMA-style flow diagram was used to document the selection process. In total, 2146 records were identified through database searching, with an additional 42 records identified through hand-searching. After removal of 312 duplicates, 1876 records were screened by title and abstract. Of these, 214 full-text articles were assessed for eligibility, and 97 were excluded for reasons including lack of focus on maternal obesity (n = 41), absence of mechanistic data (n = 33), or non-peer-reviewed or non-English status (n = 23). Ultimately, 117 studies met all inclusion criteria and were incorporated into the final narrative synthesis (Figure 1).

From each included study, data were extracted on study design, sample size and population characteristics, mechanistic domains examined, and key outcomes. Because of substantial heterogeneity across study designs, populations, mechanistic outcomes, and analytical approaches, a narrative synthesis was conducted rather than a quantitative meta-analysis. Findings are presented thematically to highlight key mechanistic pathways implicated in gestational obesity, including metabolic and inflammatory dysregulation, placental structural and functional alterations, epigenetic programming, maternal and infant microbiome interactions, and lactation- or breastfeeding-related mechanisms.

## 3. Maternal Nutrition and Fetal Development

Maternal nutrition plays a critical role in shaping fetal health and development, particularly in the context of gestational obesity. An imbalance in maternal dietary intake, whether through excessive caloric consumption or nutrient deficiencies, can lead to significant alterations in fetal development. A high-fat diet during pregnancy not only contributes to maternal obesity but also triggers epigenetic changes in the fetus, affecting metabolic pathways and increasing the risk for obesity and type 2 diabetes in later life. Moreover, both low and high-protein diets during pregnancy affect offspring health. Low-protein maternal diets increase blood pressure, body weight, and adiposity, whilst high-protein maternal diets increase body weight, blood pressure, and food efficiency and decrease energy expenditure [13,14,15,16,17]. Protein source may also influence offspring outcomes. Soy-based maternal diets increased offspring food intake, adiposity, blood pressure, homocysteine, and hypothalamic AgRP, glucose, and Homeostatic Model Assessment of Insulin Resistance (HOMA-IR) than casein-fed groups [18,19,20]. These effects suggest that protein source influences offspring phenotype, potentially through differences in digestibility, amino acid composition, and bioactive peptides encrypted in different proteins. Moreover, individual amino acids, beyond total protein content, may also influence offspring health through epigenetic mechanisms like one-carbon metabolism. Maternal low-protein diets with similar protein levels can have different effects on offspring blood pressure, possibly due to differences in methionine load and homocysteine levels [21,22,23,24,25,26,27]. Supplementing such diets with glycine or taurine can normalize blood pressure and improve insulin sensitivity and glucose regulation in offspring [28,29,30]. Additionally, the gut microbial metabolism of amino acids may impact reproduction by affecting nutrient absorption, fetal development, and the production of metabolites like nitric oxide and polyamines [31].

Evidence indicates that the developing fetus adapts its growth and development in response to the nutritional status of the mother. For instance, excessive maternal weight gain is associated with fetal macrosomia and subsequent metabolic disorders [32]. Furthermore, critical periods during gestation, marked by cellular growth and differentiation, are particularly vulnerable to the effects of maternal nutrition. Insufficient nutrient supply can lead to intrauterine growth restriction, which has profound implications for long-term cardiovascular and metabolic health outcomes in offspring [33,34]. Conversely, maternal overnutrition, characterized by high levels of free fatty acids and glucose, can induce a lipotoxic environment with detrimental effects on fetal organ systems, particularly affecting neural development and metabolic regulation [35].

Moreover, inadequate maternal intake of essential nutrients such as folate, iron, and omega-3 fatty acids has been linked to impaired fetal neurological function, potentially increasing the risk of neurodevelopmental disorders [36]. For example, iron deficiency during early gestation can lead to disruptions in gray matter structure and dendritogenesis, adversely affecting fetal cognitive function [37,38]. Moreover, calcium deficiency during pregnancy increases the risk of fetal growth restriction and preeclampsia. Vitamin D deficiency has also been correlated with abnormal brain structure and increased susceptibility to neurodevelopmental disorders like ADHD [37]. However, these associations are not consistently replicated across studies, and several factors complicate causal inference. Much of the literature is observational, where nutrient status tracks with socioeconomic context, overall diet quality, maternal adiposity, smoking, and inflammation, leaving room for residual confounding and even reverse causation (e.g., pregnancy complications altering appetite or supplement use). Exposure assessment also varies: some studies rely on FFQs, others on single-time-point biomarkers (e.g., ferritin, 25(OH)D) that shift with inflammation, hemodilution, season, and gestational age; cutoffs for “deficiency” differ across cohorts. Few analyses consider nutrient–nutrient interactions (e.g., iron–zinc, folate–B12) or gene–nutrient interactions (e.g., MTHFR for folate metabolism, FADS for LC-PUFA synthesis). Notably, randomized trials yield mixed results: DHA often shows small or domain-specific effects; iron improves maternal hematologic status but inconsistently translates to offspring cognitive gains; vitamin D trials report heterogeneous neurodevelopmental outcomes; and calcium’s preeclampsia risk reduction is clearest in low-intake populations.

The regulation of hormones influenced by maternal diet also plays a significant role in fetal development. For example, leptin regulation, influenced by maternal diet, plays a key role in fetal development. Pregnancy increases maternal leptin levels due to expanded adipose tissue and placental secretion, peaking in the second and third trimesters and dropping postpartum [39]. Maternal obesity and high-fat diets cause hyperleptinemia, impairing metabolic adaptations like insulin secretion and increasing the risk of GDM [40]. In contrast, undernutrition reduces leptin, reflecting lower fat reserves and disrupting energy balance [40,41]. Elevated maternal leptin may also alter fetal appetite regulation, increasing the offspring’s risk of obesity [42,43].

Maternal obesity may also alter how maternal diet affects offspring health. In normal-weight Wistar rats, a soy protein-based diet negatively impacted body weight, glucose metabolism, and blood pressure in male offspring compared to a casein-based diet, with sex-dependent effects [18,19,20]. However, in obese mothers, these dietary effects were largely masked, likely due to the dominant negative impact of obesity itself [44]. Similarly, differences between high- and normal-protein diets in obese dams showed limited effects, with only glucose metabolism affected [45], suggesting gestational obesity may overshadow other dietary influences.

The timing of maternal diet also critically affects offspring development. For example, periconceptional low-protein diets led to long-term metabolic changes, including increased adiposity, blood pressure, and altered gut structure, suggesting programming toward enhanced nutrient absorption and obesity risk [46,47]. Moreover, a high-protein diet during lactation, but not pregnancy, impaired muscle growth and shifted metabolism in mice [48,49], while protein or energy restriction in late gestation in goats reduced organ weights and birth weight, followed by rapid catch-up growth [49]. In minks, low post-weaning protein intake reduced growth and increased fat accumulation, regardless of maternal diet [50]. Timing also influences metabolic flexibility: in intrauterine growth restriction (IUGR) rats, responses to standard or high-energy diets varied by growth pattern, affecting insulin sensitivity and fat storage [51]. Additionally, postweaning low-protein diets induced hypertension independent of maternal diet, highlighting the critical role of early-life nutrition in shaping long-term health [52].

Nevertheless, several inconsistencies temper these conclusions. Experimental designs vary in the timing, duration, and severity of dietary manipulations, making it difficult to compare results or define critical windows of vulnerability. Moreover, some effects appear tissue-specific (e.g., muscle vs. gut vs. brain), while others are systemic, suggesting complex, organ-dependent programming pathways. The direction of impact also varies; while protein restriction is often linked to growth impairment, in some cases it promotes adaptive catch-up growth, which itself may be harmful later in life. Human studies are even less consistent, in part because controlled dietary restriction is neither ethical nor feasible; instead, observational studies rely on proxies such as maternal diet quality indices, which are subject to reporting bias and confounding. Finally, the interaction between maternal diet and postnatal nutrition introduces another layer of complexity: offspring outcomes often depend not only on prenatal exposures but also on whether early-life diets amplify or buffer these effects.

In summary, maternal nutrition plays a key role in fetal development and long-term offspring health. Both nutrient imbalances and gestational obesity can disrupt fetal programming, with effects influenced by diet composition and timing of exposure (Table 1).

## 4. Maternal Obesity and Fetal Development: Underlying Mechanisms

Maternal obesity adversely affects fetal development through metabolic, hormonal, and inflammatory pathways. It leads to chronic low-grade inflammation and disrupted glucose and lipid metabolism, impairing placental function and causing fetal overnutrition, which increases the risk of macrosomia, childhood obesity, and metabolic disorders. Key mechanisms include genetic and epigenetic alterations, such as DNA methylation and histone modification, triggered by maternal or periconceptional obesity [34,43,53]. These changes can persist into later life, affecting gene expression and disease susceptibility [43]. Inflammatory cytokines like TNF-α and IL-6 further compromise fetal tissue development and immune function [5,43,54]. Maternal metabolism also shifts during pregnancy to support fetal growth, with insulin resistance and increased glucose and fat availability in later stages. In obesity, this adaptation becomes pathological, promoting gestational diabetes, fetal hyperinsulinemia, and increased adiposity [36,42,55,56,57]. Elevated maternal glucose and lipid levels alter fetal gene expression and may impair hypothalamic development, increasing long-term risk for obesity and metabolic dysfunction [58,59,60,61].

The gut microbiome plays a crucial role in fetal development and undergoes significant changes during pregnancy. In late gestation, shifts such as increased Proteobacteria and Actinobacteria, reduced short-chain fatty acid production, and decreased microbial diversity are observed, corresponding with metabolic and immune changes and elevated inflammation [62,63]. These microbial changes are influenced by maternal BMI, gut health, and diet [64,65]. Maternal obesity is linked to gut dysbiosis, which can alter fetal metabolic programming through placental and lactational mechanisms, with possible transgenerational effects [32,36]. These findings underscore the importance of maternal metabolic health and support incorporating obesity interventions into prenatal care [36,43].

In summary, gestational obesity disrupts metabolic and hormonal pathways, increasing the risk of obesity, metabolic disorders, and neurodevelopmental issues in offspring. These effects may override the influence of specific dietary components, highlighting the complexity of maternal-fetal interactions.

### 4.1. Maternal Obesity and Fetal Programming

Maternal obesity influences offspring health primarily through fetal programming, a process in which early-life stimuli during critical developmental windows lead to lasting physiological changes [17,66]. This programming involves genetic and epigenetic modifications, such as DNA methylation, histone alterations, and non-coding RNA expression, as well as oxidative stress, inflammation, insulin resistance, placental dysfunction, and HPA axis (Hypothalamic–Pituitary–Adrenal axis) disruption [34,35]. These changes can alter gene expression related to metabolism, appetite regulation, and energy balance, increasing the risk of adiposity and impaired glucose tolerance later in life [5,36,43,67,68]. Additionally, maternal obesity negatively affects fetal brain development through inflammatory and oxidative mechanisms, elevating the risk of neurodevelopmental disorders such as ADHD and autism and contributing to long-term cognitive and psychiatric challenges [5,43,69].

#### 4.1.1. Epigenetic Modifications

Maternal obesity is strongly associated with epigenetic changes in placental, fetal, and offspring tissues, influencing long-term metabolic and developmental outcomes. Key mechanisms include DNA methylation, histone modifications, and microRNA expression [36,70]. Animal studies have shown that maternal high-fat diets can alter methylation of genes related to appetite, metabolism, and fat storage, potentially modifying offspring eating behavior [70]. In obese female mice, reduced expression of the Stella (DPPA3/PGC7) protein in oocytes suggests impaired embryonic development as a mechanism linking maternal obesity to adverse offspring outcomes [71]. These epigenetic changes can be passed across generations, contributing to intergenerational transmission of obesity and metabolic disorders [72]. However, epigenetic responses are highly tissue-specific and time-dependent, and many studies analyze only one tissue or developmental stage, limiting generalizability. Human data is less consistent than animal models, partly because maternal obesity often coexists with dietary differences, inflammation, or metabolic comorbidities such as gestational diabetes, making it difficult to isolate causal effects. Moreover, some modifications may be adaptive responses rather than pathogenic ones, raising the question of whether observed epigenetic marks truly mediate disease risk or simply reflect an altered intrauterine environment.

Gestational diabetes mellitus (GDM) also involves epigenetic modifications such as DNA methylation and microRNA-mediated gene silencing, which are influenced by maternal metabolic status [73]. Even mild hyperglycemia during pregnancy can induce persistent epigenetic changes, a concept known as “metabolic memory”, aligned with Barker’s Developmental Origins of Health and Disease (DOHAD) hypothesis [74]. These findings emphasize the importance of the intrauterine environment and suggest that improving maternal health may help reverse or mitigate epigenetic alterations, reducing long-term health risks in offspring [75]. However, not all studies consistently identify the same methylation sites or microRNAs, reflecting variability in tissue examined (placenta, cord blood, buccal cells), timing of sample collection, and analytic methods (candidate-gene vs. epigenome-wide approaches). Moreover, distinguishing the effects of GDM per se from coexisting factors, such as maternal obesity, diet, inflammation, and use of insulin or metformin, remains difficult. In many cohorts, maternal hyperglycemia clusters with other metabolic risk factors, raising the possibility of confounding.

##### DNA Methylation, Histone Modifications

DNA methylation, a key epigenetic mechanism, plays a central role in fetal programming associated with maternal obesity. Maternal obesity alters methylation patterns in critical metabolic and growth-related genes, affecting offspring health outcomes [76]. Elevated methylation of the leptin (LEP) gene promoter on the fetal side of the placenta may suppress leptin expression, disrupting fetal energy balance and growth, while hypomethylation of the adiponectin (ADIPOQ) gene on the maternal side may impair energy regulation and increase metabolic risk [76]. Genome-wide placental DNA methylation is approximately 21% higher in obese mothers, contributing to long-term susceptibility to metabolic syndromes. Disruptions in the methylation of adipokine-related genes such as LEP and ADIPOQ may elevate risks for obesity, cardiovascular disease, and metabolic syndrome [76,77]. In animal models, maternal obesity-induced hypomethylation of the Zfp423 promoter enhances adipogenic differentiation, predisposing offspring to later metabolic dysfunction [78]. These findings support the DOHAD hypothesis, linking early-life epigenetic changes to increased risk for type 2 diabetes and hypertension [79].

Importantly, these methylation patterns may be modifiable. Lifestyle interventions such as improved maternal diet and physical activity have been shown to positively influence methylation profiles, offering potential to mitigate adverse outcomes [80]. Notably, maternal weight loss after bariatric surgery has been linked to beneficial epigenetic changes in offspring, highlighting the possibility of reversing harmful programming effects [81].

Histone modifications, such as acetylation and methylation, are critical in organizing chromatin structure and gene accessibility. Maternal obesity impacts histone modifications in several tissues, including the developing brain and liver. Obesity-induced changes in histone methylation patterns near the EZH2 gene in the fetal brain impaired neural differentiation, potentially affecting offspring cognitive development [82]. Aberrant hepatic histone acetylation was observed in offspring exposed to a maternal high-fat diet, leading to altered expression of metabolic genes [83]. However, many studies are cross-sectional, with small sample sizes, and use different analytic approaches (candidate-gene vs. epigenome-wide profiling), which limits reproducibility. Moreover, the functional consequences of many observed methylation and histone changes remain unvalidated, with some potentially representing adaptive rather than pathogenic responses. Although intervention studies are promising, they are still limited, and it is not yet clear whether improving maternal health before or during pregnancy fully normalizes offspring epigenetic profiles.

##### Non-Coding RNA Expression

Non-coding RNAs (ncRNAs), including microRNAs (miRNAs) and long non-coding RNAs (lncRNAs), are key post-transcriptional regulators of gene expression and are significantly influenced by maternal obesity. Altered expressions of lncRNAs such as H19 and MALAT1 in obese pregnancies can disrupt fetal stem cell differentiation and developmental processes, increasing susceptibility to metabolic disorders later in life [43,82,84]. These changes can persist beyond gestation through epigenetic modifications, aligning with the developmental origins of health and disease (DOHaD) hypothesis. Maternal high-fat diets have also been shown to modulate ncRNA expression, contributing to offspring risk for obesity and type 2 diabetes [85]. Given their sensitivity to the intrauterine environment, ncRNAs are emerging as both biomarkers and potential therapeutic targets [86].

MiRNAs, a major class of ncRNAs, regulate gene expression by binding to mRNAs, leading to their degradation or translational repression [70]. Maternal obesity alters miRNA profiles in offspring, impacting essential processes such as growth, metabolism, and inflammation [87,88,89,90]. For instance, dysregulation of miRNAs involved in lipid metabolism and inflammatory pathways contributes to adipogenesis and metabolic dysfunction. Epigenetic mechanisms such as DNA methylation and histone modifications further regulate miRNA expression. Notably, altered expression of miR-143 and miR-145 in obese pregnancies affects fatty acid metabolism, increasing the risk of metabolic disorders [43]

These miRNA changes have been observed in various offspring tissues, including liver, heart, and muscle [70]. In mouse models, maternal high-fat diets suppressed miRNAs critical for lipid metabolism and the regulation of Igf2, contributing to impaired developmental timing and metabolic outcomes. Such changes may also underline the programming of appetite and energy regulation systems [91]. Beyond metabolism, miRNAs modulate neurodevelopment, with altered expression patterns affecting neurogenesis, synaptic plasticity, and inflammatory responses in the developing brain, further linking maternal obesity to long-term cognitive and neurodevelopmental risks [70]. However, maternal obesity frequently co-occurs with gestational diabetes, altered diets, and systemic inflammation, making it difficult to separate direct effects of obesity from these confounders. Moreover, the functional validation of identified miRNAs is also limited: many associations are correlative, with few mechanistic studies confirming causality.

#### 4.1.2. Maternal Obesity and Fetal Hyperinsulinemia

Although insulin is essential for fetal growth [92], elevated fetal insulin levels, commonly seen in perinatal hyperinsulinemia, are associated with an increased risk of obesity and glucose intolerance later in life, particularly in offspring of mothers with diabetes or mild glucose intolerance [93,94,95,96,97]. The fetal insulin hypothesis suggests that low birth weight and insulin resistance may share a genetic origin, emphasizing insulin’s critical role in fetal development [92,98]. Excess fetal insulin can disrupt neuroendocrine development, especially in the hypothalamus, a region highly sensitive to hormonal cues during gestation [99]. In gestational obesity, elevated insulin levels may cause permanent changes in hypothalamic structures such as the ventromedial nucleus (VMN), impairing energy regulation and metabolism [97,99,100,101]. These alterations may lead to leptin and insulin resistance, increased expression of orexigenic peptides like NPY and galanin, and a heightened risk of long-term obesity and hyperinsulinemia [60,61].

Gestational diabetes mellitus (GDM) further increases the risk of fetal hyperglycemia and hyperinsulinemia, contributing to adverse metabolic programming in offspring compared to those born to non-diabetic mothers [102]. As described earlier, fetal hyperinsulinemia can impair the development of hypothalamic systems regulating food intake and body weight, resulting in long-lasting susceptibility to obesity [103]. Additionally, pre-pregnancy maternal hyperinsulinemia, inflammation, and oxidative stress contribute to early placental and fetal dysfunction [53,57]. Importantly, even in the absence of clinical GDM, maternal obesity can lead to mild hyperglycemia that still promotes fetal hyperinsulinemia and its associated risks [104].

#### 4.1.3. Maternal Obesity and Oxidative Stress

Oxidative stress is increasingly recognized as a key mechanism linking maternal obesity to adverse fetal programming outcomes. Excessive production of reactive oxygen species (ROS) disrupts cellular functions and regulatory pathways in fetal tissues, particularly affecting cardiovascular and metabolic development [35,105]. Maternal obesity contributes to oxidative stress through elevated levels of glucose and free fatty acids, which impair antioxidant defenses and promote lipid peroxidation, potentially resulting in long-term metabolic dysfunction [36].

Oxidative stress arises from an imbalance between ROS production and antioxidant capacity, leading to cellular damage and impaired developmental processes. Conditions common in obesity, such as inflammation and hypoxia, can exacerbate this imbalance during pregnancy, with significant implications for fetal growth and organ development [35]. Vulnerable tissues like the brain and heart are especially affected during critical developmental windows.

The placenta plays a central role in this context, as it both reflects the oxidative state of the maternal environment and regulates fetal exposure to oxidative insults. Oxidative stress impairs placental function, compromising nutrient and gas exchange, and contributing to fetal growth restriction [106]. It also influences gene expression and cellular signaling pathways, increasing the offspring’s risk for insulin resistance, dysregulated lipid metabolism, and other components of metabolic syndrome. Maternal conditions such as diabetes and inflammation may further amplify oxidative stress, predisposing the offspring to neurodevelopmental and metabolic disorders later in life [70]. However, biomarkers of oxidative stress (e.g., malondialdehyde, 8-iso-prostaglandin F2α, antioxidant enzyme activity) vary widely across studies, and their reliability is affected by differences in sample type (placenta, cord blood, maternal plasma) and timing of collection. Moreover, heterogeneity in populations, diets, and coexisting conditions can be factors resulting in inconsistent results.

#### 4.1.4. Maternal Obesity and Placental Alterations

The placenta, as a critical interface between maternal and fetal systems, also modulates the effects of maternal metabolic health on fetal programming. It regulates the transport of nutrients and metabolites while simultaneously mediating the maternal hormonal milieu [42].

For instance, increased placental production of leptin and alterations in amino acid transporter expression during maternal obesity can enhance fetal growth, contributing to macrosomia and later obesity risks [42,105]. In contrast, insufficient nutrients due to metabolic dysregulation can lead to fetal growth restriction, with both scenarios having long-term consequences for metabolic health in offspring [33]. Placental dysfunction is also an influencing factor in developmental programming [107]. Excess maternal adiposity affects placental structure and function, leading to altered nutrient transport, hypoxia, and oxidative stress, which can impact fetal growth and development [108,109,110].

#### 4.1.5. Maternal Obesity and HPA Axis Alteration

Alteration in the hypothalamic–pituitary–adrenal (HPA) axis may result in a higher risk of obesity in offspring [111]. Stimulation of the maternal hypothalamic–pituitary–adrenal (HPA) axis may play a key role in altering the in utero environment, potentially contributing to cardiometabolic diseases in the offspring later in life [112]. Maternal nutrition can alter the expression of hypothalamic genes, potentially increasing fetal and neonatal energy intake. Epigenetic modifications may contribute to the global rise in obesity and other metabolic disorders, as their effects can be transmitted across generations [68]. In addition, maternal obesity and stress can also dysregulate fetal HPA axis development, increasing fetal exposure to glucocorticoids [113,114]. This can lead to altered stress responses, increased susceptibility to obesity, neurodevelopmental disorders, and cardiometabolic diseases in the offspring [112,115,116,117,118,119].

### 4.2. Maternal Obesity and Metabolism

Maternal metabolism also plays a crucial role in the process of fetal development, particularly in the context of obesity. Obesity in general can lead to hyperinsulinemia, possibly due to low-grade inflammation and endotoxemia [57]. Maternal obesity leads to a hyperglycemic and hyperlipidemic intrauterine environment, which asserts a significant influence on the developing fetus [36,42]. It also increases insulin resistance and significantly raises the risk of gestational diabetes mellitus and altered fat oxidation (GDM) [57,120,121,122], which can lead to an increased fetal glucose exposure, hyperinsulinemia, and adiposity, which predispose the offspring to obesity and insulin resistance [123,124]. This can result in an increased risk of early-onset type 2 diabetes and metabolic syndrome in childhood and adulthood [58,59].

Elevated levels of glucose and free fatty acids have programming effects and predispose offspring to obesity and metabolic disorders later in life [32,36]. Specifically, these metabolic disturbances can lead to alterations in the fetal expression of genes associated with adipogenesis, insulin sensitivity, and energy metabolism [36,70]. A review by Nelson et al. [122] suggested that gestational-related fat is accrued in the trunk of the woman and is a mixture of visceral fat, which is associated with lower insulin sensitivity, lipid dysfunction and high blood pressure as well as subcutaneous fat [122]. Prior studies have shown that there is a 50–80% increase in fat oxidation during pregnancy as well in response to glucose that could lead to hyperlipidemia [125]. Hyperlipidemia is thought to be exaggerated in obese women, which could lead to vascular conditions and increase cardiovascular risks [126].

Maternal obesity may also alter fat supply to the fetus. Stored long-chain polyunsaturated fatty acids (LCPUFAs) in fetal tissue support early postnatal development [127]. In normal conditions, in late pregnancy, fetal fat deposition peaks, with n-3 LCPUFA needs often exceeding maternal dietary intake. Maternal fat stores and placental adaptations help maintain fetal supply [128]. Maternal obesity and excessive gestational weight gain may alter fatty acid (FA) transfer to the fetus and infant, reducing n-3 and increasing n-6 PUFA levels in circulation and breast milk [127]. These changes promote inflammation and oxidative stress, affecting fetal organs and raising the risk of obesity, neurodevelopmental disorders, asthma, and cancer [127].

While strong mechanistic links between maternal obesity, hyperglycemia, hyperlipidemia, and fetal metabolic programming are supported, inconsistencies remain. Human studies vary widely in how metabolic exposures are measured, fasting vs. postprandial glucose, lipid fractions, or insulin sensitivity indices, and at which stage of pregnancy. This makes it difficult to identify critical windows of exposure. Moreover, inter-individual variation in placental function and fatty acid transport adds further complexity. For LCPUFAs specifically, studies differ in whether maternal obesity reduces, increases, or has no effect on fetal supply, likely reflecting differences in dietary background and genetics (e.g., FADS gene polymorphisms) (Table 2).

## 5. Maternal Obesity and Breastfeeding

Breastfeeding may be more challenging for mothers who are overweight or obese due to chronic low-grade inflammation, which can adversely affect neonatal outcomes [129]. Studies have consistently shown lower breastfeeding initiation rates among women with obesity. A meta-analysis by Turcksin et al. [130] found that women with a BMI ≥30 were approximately 15–20% less likely to initiate breastfeeding compared to women with a BMI in the normal range (18.5–24.9). This finding has been corroborated by numerous population-based studies, including a large cohort study of over 4000 women by Kair and Colaizy [131], which demonstrated that women with obesity were 1.5 times more likely to never initiate breastfeeding compared to normal-weight counterparts, even after adjusting for sociodemographic factors.

### 5.1. Challenges in Breastfeeding for Women with Obesity

Women with obesity tend to breastfeed for shorter durations and are less likely to breastfeed exclusively. The National Immunization Survey (NIS) found that only 39% of obese mothers were still breastfeeding at 6 months, compared to 53% of mothers with normal BMI [132].

#### 5.1.1. Physiological Factors

Delayed onset of lactogenesis II (the stage when milk volume increases dramatically postpartum) is more common in women with obesity. Typically expected by 72 h after delivery, delayed lactogenesis is associated with increased infant supplementation and a reduced likelihood of sustained breastfeeding [133]. Mechanistically, this delay appears to result from multiple interrelated physiological alterations. Insulin resistance, which is prevalent in obesity, disrupts the hormonal cascade necessary for lactation [134], while elevated inflammatory cytokines such as TNF-α and IL-6 may impair prolactin signaling and milk synthesis [135]. Altered endocrine function, including lower circulating oxytocin levels, negatively affects milk ejection reflexes [136]. Furthermore, excessive adiposity can impair mammary gland differentiation during pregnancy by disrupting the delicate balance of estrogen, progesterone, and prolactin—hormones critical to successful lactogenesis [137]. Animal models and imaging studies in humans suggest that the mammary gland microstructure may be compromised in obesity, reducing milk-secreting alveolar cells and ducts [138]. Moreover, maternal obesity alters milk composition, with higher concentrations of pro-inflammatory biomarkers (SAA, CRP, TNF-α, IL-8, and IFN-γ) than anti-inflammatory markers like IL-10 [129,139]. Recent research by Panagos et al. [140] has also revealed that maternal obesity is associated with altered fatty acid profiles in breast milk, with decreased levels of polyunsaturated fatty acids (PUFAs), which are crucial for infant neurodevelopment. A longitudinal study by Nommsen-Rivers [141] demonstrated that for each 1-unit increase in maternal BMI, the odds of delayed lactogenesis increased by 6%, underscoring the dose-dependent relationship between obesity and lactation difficulties.

#### 5.1.2. Psychosocial Factors

Negative body image and low maternal self-efficacy are more common in mothers with obesity and strongly associated with reduced breastfeeding success [142]. Many women feel uncomfortable breastfeeding in public or even around family, contributing to early weaning [142]. Obesity increases the risk of perinatal depression, which independently predicts lower breastfeeding rates [143]. Depression can interfere with bonding, motivation, and the physical effort required to breastfeed, particularly during the demanding newborn period [144]. Women with obesity more frequently report concerns about low milk supply, whether perceived or actual [145], and uncertainty about milk transfer or infant satiety can prompt early formula supplementation [146,147]. A qualitative study found that women with obesity experienced more physical discomfort with positioning and latching due to larger breast size, contributing to early weaning [148]. Reduced breastfeeding success is also partly mediated by lower breastfeeding self-efficacy scores [149]. Additionally, healthcare provider bias may play a role, as women with obesity report receiving less breastfeeding encouragement and support than normal-weight mothers [149].

#### 5.1.3. Mechanical and Practical Considerations

Women with obesity face unique mechanical challenges during breastfeeding that are often underrecognized in clinical settings. Larger breast size can make proper positioning and latch more difficult, potentially leading to nipple trauma and pain [147]. Additionally, finding comfortable breastfeeding positions that accommodate larger body sizes may require specialized support and education. Babendure et al. [150] noted that the standard breastfeeding positions taught in prenatal classes may not be practical for women with higher BMIs, necessitating tailored approaches. Moreover, difficulties with mobility and postpartum recovery, particularly following cesarean deliveries (which are more common in women with obesity), can further complicate early breastfeeding efforts [151,152].

### 5.2. Maternal Obesity and Breast Milk Microbiota

The impact of maternal obesity extends beyond breastfeeding challenges, influencing the composition of breast milk microbiota (BMM), a critical seeding source for the infant gut microbiome. The BMM contains a diverse array of bacteria, including *Staphylococcus*, *Streptococcus*, *Lactobacillus*, and *Bifidobacterium*, which contribute to the immunological and metabolic maturation of the infant gut. Factors such as delivery-related antibiotic exposure, breastfeeding practices, and maternal lifestyle can shape the bacterial composition of human milk [153]. Obese mothers exhibit altered BMM profiles, with increased levels of *Staphylococcus* and *Prevotella* in colostrum, potentially contributing to early-life inflammation and unfavorable metabolic programming [154]. This finding is particularly significant as it suggests that maternal obesity may affect infant health beyond simply nutritional content of breast milk.

Recent evidence indicates that the diversity and composition of BMM are significantly modulated by maternal body mass index (BMI), mode of delivery, hormonal milieu, and systemic inflammation [155]. Higher maternal BMI has been associated with lower microbial diversity in breast milk and a reduced presence of beneficial taxa such as *Bifidobacterium* and *Lactobacillus*, both of which are important for promoting anti-inflammatory responses in the neonate [156,157]. A comprehensive metagenomic analysis by Moossavi et al. [158] revealed that maternal obesity is associated with a distinct microbial signature in human milk, characterized by decreased levels of *Bifidobacterium* and increased abundances of potentially pathogenic bacteria. These alterations persisted even after controlling for mode of delivery and antibiotic exposure, suggesting a direct effect of maternal metabolism on milk microbiota composition. Similarly Karami et al. [159] reported that breast milk from normal-weight mothers contained higher *Bifidobacterium* spp. than milk from mothers with obesity. Higher current and pre-pregnancy weight were linked to lower levels of *Actinobacteria*, *Firmicutes*, and *Lactobacillus*, while cesarean delivery further reduced *Bacteroidetes* and *Firmicutes*.

A longitudinal study found that the influence of maternal obesity on BMM persists across lactation stages, with sustained enrichment in pro-inflammatory genera and reductions in beneficial commensals [160]. Moreover, the altered BMM of obese mothers was linked to lower secretory IgA levels, compromising immune protection and mucosal development in infants. Kumar et al. [161] demonstrated that these changes in milk microbiota composition correlate with altered infant gut microbiome development at 3 and 6 months of age, suggesting that maternal obesity-associated dysbiosis may have long-lasting effects on offspring gut health.

While breastfeeding remains the gold standard for infant nutrition and microbiome establishment, its capacity to fully counteract maternal obesity-associated dysbiosis is limited. Raspini et al. [162] highlight that although breastfeeding promotes microbial diversity in infants, its effectiveness may be blunted in the context of maternal metabolic derangements. These findings suggest that early interventions targeting maternal weight and inflammation may be necessary to optimize BMM composition and downstream infant outcomes. Interestingly, a study by Garcia-Mantrana and Collado [163] found that maternal dietary patterns during lactation, particularly adherence to Mediterranean diet principles, partially mitigated the negative impact of maternal obesity on milk microbiota, suggesting a potential avenue for intervention.

Additionally, it has been proposed that microbial alterations in breast milk are not only reflective of maternal gut dysbiosis but also influenced by the entero-mammary pathway, where maternal gut bacteria are trafficked to the mammary gland via dendritic cells [164]. This highlights the importance of maternal gut health in shaping milk microbiota, with potential translational interventions such as maternal probiotics or dietary modification during lactation. Recent work by Cabrera-Rubio et al. [165] demonstrated that probiotic supplementation during pregnancy and lactation modified breast milk microbiota composition in women with obesity, increasing the abundance of beneficial bacteria and potentially improving infant gut colonization patterns. Furthermore, Williams et al. [166] found that prebiotic supplementation during lactation improved the glycemic profile of mothers with obesity and positively influenced the microbial composition of their breast milk, suggesting a promising interventional approach.

### 5.3. Implications for Infant Development and Health

The consequences of obesity-associated alterations in breastfeeding patterns and milk composition extend to infant development and health trajectories. Infants of mothers with obesity who breastfeed show different growth patterns compared to those born to normal-weight mothers [167,168]. Eriksen et al. [167] demonstrated that despite the protective effect of breastfeeding against childhood obesity, this protection is attenuated when the breastfeeding mother has obesity. This suggests that qualitative aspects of breast milk may be as important as the act of breastfeeding itself.

The human milk oligosaccharide (HMO) profile, crucial for infant gut development and immunity, is also altered in mothers with obesity. Bardanzellu et al. [169] found that obesity is associated with lower concentrations of specific HMOs that promote the growth of beneficial bacteria like Bifidobacterium. This may partially explain the differences in gut microbiome development observed in infants of mothers with obesity, even when exclusively breastfed.

Moreover, the inflammatory profile of breast milk from mothers with obesity may contribute to altered immune programming in infants. Whitaker et al. [139] found that infants exclusively breastfed by mothers with obesity showed distinct inflammatory cytokine profiles compared to those breastfed by normal-weight mothers, potentially setting the stage for altered immune responses later in life. These findings underscore the complex interplay between maternal metabolism, milk composition, and infant development.

The findings are synthesized and summarized in Table 3. 

## 6. Study Strengths and Limitations

This review has several key strengths. First, it provides a comprehensive synthesis of interdisciplinary evidence, incorporating clinical, mechanistic, and epidemiological studies across human and animal models. By examining maternal obesity from multiple perspectives, including metabolic, epigenetic, placental, microbiome, and breastfeeding pathways, the manuscript offers a holistic and integrative framework for understanding intergenerational health effects. The inclusion of recent literature (2022–2025) enhances the timeliness and relevance of the review, ensuring that readers are exposed to the latest findings. Another important strength is the emphasis on emerging mechanisms, particularly breast milk microbiota, which remains underexplored in maternal-infant research. The structured methodology, involving predefined inclusion and exclusion criteria, improves transparency and reproducibility, while the narrative synthesis approach allows for integration of heterogeneous findings that would not be amenable to meta-analysis. Finally, the review highlights gaps in knowledge and clinical implications, making it not only a summary of evidence but also a forward-looking resource for researchers and practitioners.

Several limitations should be acknowledged. First, as a narrative review, the synthesis is subject to potential selection and reporting bias, despite structured search methods. The heterogeneity of study designs, populations, and outcome measures limit direct comparability across studies, and conclusions should be interpreted with caution. Many mechanistic insights are drawn from animal studies, which may not fully capture the complexity of human physiology and maternal-fetal interactions. Furthermore, definitions of maternal obesity vary widely, ranging from pre-pregnancy BMI to gestational weight gain, introducing inconsistency across the literature. Confounding factors such as socioeconomic status, maternal diet quality, physical activity, and genetic background were not consistently adjusted for in the primary studies, which may bias associations between maternal obesity and offspring outcomes. Additionally, most studies are short- to medium-term, with limited longitudinal follow-up into adolescence or adulthood, restricting the ability to fully capture intergenerational effects. Finally, while the review covers multiple mechanistic domains, some areas, such as nutrient-specific effects, sex-specific offspring outcomes, and intervention strategies, remain incompletely understood, underscoring the need for further research.

## 7. Conclusions

**Figure 2:**
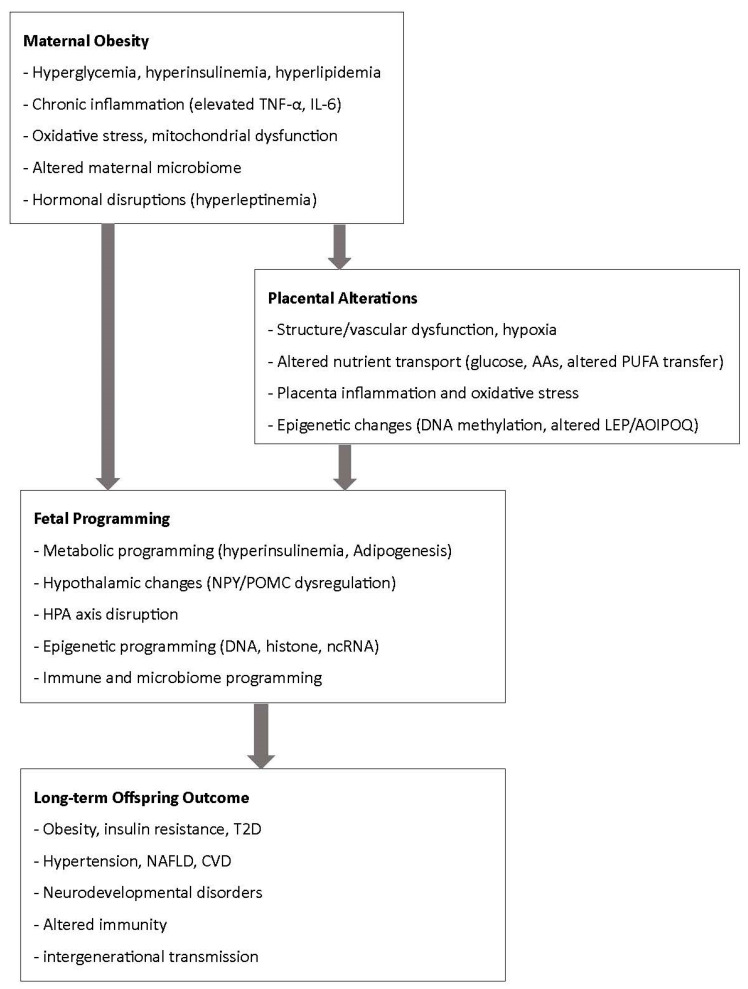
Summary of mechanistic pathways from maternal obesity to fetal programming.

This narrative review underscores the impact of maternal obesity on both maternal and offspring health. Maternal obesity exerts profound and multifaceted effects on pregnancy outcomes, fetal programming, and long-term offspring health. Mechanistic evidence highlights the central role of metabolic dysregulation, inflammation, oxidative stress, epigenetic reprogramming, and alterations in the gut and breast milk microbiota in shaping developmental trajectories. These pathways converge to increase risks for macrosomia, childhood obesity, cardiometabolic disease, and neurodevelopmental disorders, underscoring the intergenerational consequences of maternal metabolic health (Figure 2).

Despite these challenges, the findings also highlight opportunities for prevention and intervention. Optimizing maternal nutrition, addressing obesity prior to and during pregnancy, and exploring targeted strategies such as dietary modification, microbiome-directed therapies, and lifestyle interventions may help mitigate adverse outcomes. Integrating maternal health interventions into prenatal care, with attention to both biological and psychosocial determinants, is critical for breaking the cycle of obesity and improving health trajectories for both mothers and their children (Figure 2).

## 8. Future Directions

The complex relationship between maternal obesity and offspring health necessitates further research in several key areas. While substantial progress has been made in understanding the mechanisms linking maternal obesity to adverse maternal and offspring outcomes, several gaps remain that warrant further investigation. First, disentangling the relative contributions of metabolic, inflammatory, epigenetic, and microbiome-mediated pathways remains a priority, as these mechanisms are often interdependent and vary across gestational stages. Multi-omics approaches integrating genomics, epigenomics, metabolomics, and microbiome profiling could provide a more comprehensive picture of these complex interactions. Second, longitudinal studies with extended follow-up are needed to better characterize the persistence of obesity-related programming effects into adolescence and adulthood, with attention to sex-specific differences in outcomes. Third, greater emphasis should be placed on translational research that moves beyond mechanistic understanding toward clinical interventions. Lifestyle modification, dietary optimization, targeted supplementation, and microbiome-directed therapies should be rigorously tested in controlled trials to determine their efficacy in reducing intergenerational transmission of obesity and metabolic disease.

Finally, equity-focused research is urgently needed to ensure interventions are culturally sensitive, accessible, and feasible for diverse populations. Addressing maternal obesity within the broader context of social determinants of health, including access to nutritious food, healthcare, and education, will be essential for achieving meaningful improvements in maternal and child health worldwide.

## Figures and Tables

**Table 1:** Summary of Maternal Nutrition and Fetal Development.

Nutritional Factor	Offspring Effects	Risk Estimates/Evidence	References
High-fat maternal diet	Epigenetic changes; ↑ risk of obesity and T2D	Strong evidence from animal and human studies; ↑ lifelong obesity and T2D risk	[13,14,15,16,17]
Low-protein maternal diet	↑ BP, body weight, adiposity	Animal models; consistent ↑ BP and adiposity	[13,14,15,16,17]
High-protein maternal diet	↑ Body weight, BP, food efficiency; ↓ energy expenditure	Animal studies; moderate evidence; ↑ metabolic dysfunction	[13,14,15,16,17]
Protein source (soy vs. casein)	Soy diet → ↑ adiposity, BP, HOMA-IR vs. casein	Rat studies; strong sex-dependent effects; clinical translation limited	[18,19,20]
Individual amino acids (methionine, glycine, taurine)	Methionine load ↑ BP; Glycine/taurine supplementation improves glucose regulation	Animal studies show normalization with supplementation; mechanistic support strong	[21,22,23,24,25,26,27,28,29,30]
Gut microbial metabolism of amino acids	Impacts reproduction, nutrient absorption, fetal growth	Emerging evidence; microbial shifts linked to reproduction/fetal growth	[31]
Excessive maternal weight gain	Fetal macrosomia; ↑ risk of metabolic disorders	Clinical evidence; strong association with macrosomia and metabolic risk	[32]
Nutrient deficiencies (folate, iron, omega-3, calcium, vitamin D)	Impaired neurological development, IUGR, preeclampsia, ADHD risk	Human and animal data; deficiencies linked to impaired neurodevelopment and IUGR	[36,37,38]
Leptin regulation (maternal diet influence)	Hyperleptinemia → GDM risk; altered fetal appetite regulation	Strong evidence; maternal leptin dysregulation linked to GDM and offspring obesity	[39,40,41,42,43]
Maternal obesity and protein source effects	Obesity masks protein source effects; limited impact beyond glucose metabolism	Animal models; obesity is dominant factor masking dietary differences	[44,45]
Timing of maternal diet (periconception, gestation, lactation, postweaning)	Periconception low protein → long-term obesity risk; lactation diet alters muscle growth; late restriction → low birth weight + catch-up growth	Timing critical; strong animal evidence; human data emerging	[46,47,48,49,50,51,52]

**Table 2:** Summary of Maternal Obesity and Fetal Development: Underlying Mechanisms.

Mechanism	Description	Offspring Outcomes	Risk Estimates/Evidence	References
Epigenetic Modifications (DNA methylation, histone, ncRNA)	Maternal obesity alters DNA methylation, histone modifications, and ncRNA expression in placenta and fetal tissues. Alters metabolic and appetite-regulating genes; intergenerational effects possible.	↑ Risk of obesity, T2D, CVD, cognitive/neurodevelopmental disorders	Strong evidence from human and animal studies; genome-wide methylation ↑21% in obese mothers; intergenerational risk supported	[34,35,36,37,38,39,40,41,42,43,67,68,69,70,71,72,73,74,75,76,77,78,79,80,81,82,83,84,85,86,87,88,89,90]
Fetal Hyperinsulinemia	Elevated maternal glucose/insulin leads to fetal hyperinsulinemia, hypothalamic alterations, leptin/insulin resistance, increased risk of obesity and metabolic disease.	↑ Risk of obesity, hyperinsulinemia, metabolic dysfunction	Well-documented in human and animal studies; consistent ↑ risk of obesity, T2D	[92,93,94,95,96,97,98,99,100,101,102,103,104]
Oxidative Stress	Excess glucose/FFAs increase ROS, impair antioxidant defenses, damage fetal cardiovascular, neural, and metabolic development.	↑ Cardiovascular risk, metabolic dysfunction, neurodevelopmental issues	Strong mechanistic support; animal/human data link ROS to placental/fetal dysfunction	[35,36,105,106,107,108,109,110]
Placental Alterations	Obesity alters placental structure, nutrient transport, hormone production (e.g., leptin), hypoxia, oxidative stress → macrosomia or growth restriction.	Macrosomia, obesity, growth restriction, long-term metabolic dysfunction	Robust evidence; placental dysfunction strongly associated with macrosomia and IUGR	[42,105,106,107,108,109,110]
HPA Axis Alteration	Maternal obesity/stress alters fetal HPA axis, increasing glucocorticoid exposure; long-term risk of obesity, stress disorders, cardiometabolic disease.	↑ Risk of obesity, neurodevelopmental disorders, stress sensitivity, cardiometabolic disease	Animal and clinical data support; strong mechanistic plausibility for long-term risks	[111,112,113,114,115,116,117,118,119]
Maternal Metabolism (Hyperglycemia, Hyperlipidemia, Insulin Resistance)	Obesity increases insulin resistance, GDM risk, altered FA oxidation, hyperglycemia, hyperlipidemia → fetal adiposity, obesity, early T2D, metabolic syndrome.	↑ Obesity, insulin resistance, metabolic syndrome, CVD risk	Extensive clinical evidence; GDM and hyperlipidemia linked to ↑ offspring obesity and T2D	[36,42,57,120,121,122,123,124,125,126]
Gut Microbiome Changes	Maternal obesity linked to gut dysbiosis; altered microbial composition reduces SCFA production, increases inflammation, affects fetal immune/metabolic programming.	Altered metabolic and immune programming; possible transgenerational effects	Emerging evidence; growing support from human cohorts and mechanistic studies	[32,36,62,63,64,65]

**Table 3:** Summary of Maternal Obesity and Breastfeeding.

Challenge/Mechanism	Description	Infant/Maternal Outcomes	Risk Estimates/Evidence	References
Breastfeeding initiation	Women with obesity are ~15–20% less likely to initiate breastfeeding compared to normal-BMI women.	Lower breastfeeding initiation rates, higher formula use.	1.5× higher risk of never initiating breastfeeding.	[130,131]
Breastfeeding duration/exclusivity	Obese mothers breastfeed for shorter durations; lower exclusivity at 6 months.	Reduced sustained breastfeeding, higher supplementation.	39% of obese mothers vs. 53% of normal-BMI mothers breastfeeding at 6 months.	[132]
Delayed lactogenesis II	Obesity linked to delayed onset (>72 h) of milk production due to insulin resistance, inflammation, and endocrine disruption.	Increased infant supplementation, early cessation of breastfeeding.	6% increased odds of delayed lactogenesis per BMI unit increase.	[133,134,135,136,137,138,139,140,141]
Altered milk composition	Higher pro-inflammatory cytokines, altered PUFA profile, reduced beneficial nutrients in breast milk.	Potential impairment of infant neurodevelopment, altered immune signaling.	Decreased PUFAs, elevated inflammatory markers in milk.	[129,139,140]
Psychosocial factors	Negative body image, lower self-efficacy, higher depression rates, provider bias, perceived low milk supply.	Early cessation of breastfeeding, reduced exclusivity.	Self-efficacy partially mediates obesity–breastfeeding relationship.	[142,143,144,145,146,147,148,149,150]
Mechanical/practical challenges	Larger breast size complicates latching and positioning; increased C-section recovery barriers.	Higher nipple trauma, pain, early weaning.	Need for tailored breastfeeding positions and clinical support.	[148,151,152,153]
Breast milk microbiota (BMM)	Obesity alters BMM, increasing Staphylococcus/Prevotella, reducing Bifidobacterium/Lactobacillus diversity.	Infant gut dysbiosis, altered immune programming, higher inflammation risk.	Persistent dysbiosis across lactation; reduced IgA in obese mothers’ milk.	[154,155,156,157,158,159,160]
Maternal diet/probiotic interventions	Mediterranean diet, probiotics, and prebiotics can partially reverse obesity-related BMM alterations.	Improved maternal metabolism, breast milk microbiota, and infant gut colonization.	Probiotic supplementation increased beneficial bacteria in milk.	[162,163,164,165]
Infant outcomes	Obesity-associated breast milk changes attenuate breastfeeding’s protection against obesity.	Altered growth, immune development, and increased long-term obesity risk.	Differences in HMO and cytokine profiles linked to metabolic programming.	[139,166,167,168]

## Data Availability

This is a narrative review, and therefore, no new data were created in this study. Data sharing does not apply to this article.
